# Analysis of Phenotypic Variability in Natural Populations of *Cereus fernambucensis* Lem. (Cactaceae)

**DOI:** 10.3390/biology14121702

**Published:** 2025-11-29

**Authors:** João Henrique Constantino Sales Silva, Joyce Naiara da Silva, Aline das Graças Souza, Naysa Flávia Ferreira do Nascimento, Edna Ursulino Alves

**Affiliations:** 1 Postgraduate Program in Agronomy, Federal University of Paraíba, University Campus II, Areia 58397-000, PB, Brazil; joicenaiara@hotmail.com (J.N.d.S.); alinedasgracas@yahoo.com.br (A.d.G.S.); naysa.flavia@academico.ufpb.br (N.F.F.d.N.); ursulinoalves@hotmail.com (E.U.A.); 2 Department of Plant Science and Environmental Sciences, Center for Agrarian Sciences, Federal University of Paraíba, University Campus II, Areia 58397-000, PB, Brazil

**Keywords:** adaptive potential, biodiversity conservation, biometrics, coastal vegetation, columnar cactus, genetic variability

## Abstract

Genetic diversity is essential for species adaptation, evolution, and conservation and is a determining factor in plants of ecological and agronomic interest, such as *Cereus fernambucensis* (Cactaceae). This study evaluated phenotypic variability and the relative importance of morphophysiological traits in two natural populations of the species located in *restinga* areas in Paraíba, Brazil. Twenty subpopulations were analyzed in a completely randomized design with four replicates and 15 traits. Genetic divergence was estimated by the Mahalanobis distance and grouped via the Tocher method, whereas the relative contribution of the characters was determined via the Singh method, which is associated with a selection index to identify promising genotypes. The results indicated the formation of nine distinct groups, with the mean germination time and germination speed index being the most relevant variables for differentiation. Subpopulations 4 and 13 (area I) and 15, 16, 17, and 18 (area II) stood out as strategic genetic reservoirs for conservation and improvement. Thus, this study reinforces the importance of maintaining the genetic diversity of *C*. *fernambucensis*, especially in the face of habitat fragmentation, ensuring evolutionary resilience, enriching germplasm banks, and supporting programs for the sustainable use and valorization of the genetic resources of columnar cacti in coastal ecosystems.

## Simple Summary

Genetic diversity is the basis for species survival and adaptation as it ensures that plants and animals are able to cope with changes in the environment. In the case of *Cereus fernambucensis*, a cactus found in *restinga* areas (coastal dune vegetation), understanding phenotypic diversity helps to choose which plants have the greatest potential for conservation, propagation, and sustainable use. This study analyzed two natural populations of this species in Paraíba, Brazil, observing different traits of the plants, fruits, seeds, and seedlings. Twenty groups of plants were evaluated, and based on various comparisons, nine distinct sets were identified. Among the traits that most differentiated the groups were the mean germination time (MGT) and germination speed index (GSI) of the seeds. Some subpopulations, from areas called I and II, have proven to be important genetic reservoirs; that is, they have plants with great potential to ensure the conservation and improvement of the species. These results highlight the need to preserve the diversity of *C*. *fernambucensis*, especially in light of the loss of natural habitat, ensuring its long-term survival. In addition, this work contributes to conservation strategies, the creation of seed banks, and the appreciation of coastal cacti as a fundamental part of biodiversity.

## 1. Introduction

Cacti are key components of arid and semiarid ecosystems in the Americas and exhibit wide genetic diversity and morphophysiological adaptation to various environmental conditions [1]. The genus *Cereus* Mill. (Cactaceae; Cereeae) comprises approximately 30 predominantly South American species, with the Brazilian Cerrado being the ecosystem most likely to be the ancestral dispersal site of this group [2,3]. The diversification of the genus *Cereus* was driven by climatic instabilities that occurred millions of years ago, involving ecosystem changes and biogeographic transitions, which resulted in phenotypic changes over time [3].

The Brazilian Atlantic Forest (BAF) is considered a biodiversity hotspot and harbors high levels of cactus endemism, including the monophyletic group *Cereus fernambucensis* Lem., *Cereus sericifer* F. Ritter, and *Cereus insularis* Hemsley, which form clade A2 [4]. The species *C*. *fernambucensis* (beach *mandacaru*) is a shrubby, decumbent plant with succulent branches, white nocturnal flowers, and fleshy dehiscent fruits with numerous small black seeds [5]. This cactus has a wide latitudinal distribution in the continental *restinga*, growing on dunes and rocky beaches [6], making it a suitable model for investigations into phenotypic variability across relatively drier environments in the BAF.

*C*. *fernambucensis* has CAM photosynthetic metabolism, produces edible fleshy fruits that are magenta in color with white endocarp, rich in nutrients and has high antioxidant activity, although these fruits are not yet commercially exploited [7]. This species reproduces sexually and vegetatively, and seed propagation is essential for genetic diversity. Although it is self-compatible, cross-pollination is most effective, with sphingids at night and bees during the day as pollinators. The flowers also provide shelter and food for various insects, highlighting ecological interactions beyond pollination [8]. These characteristics make the beach *mandacaru* a relevant species, both economically and ecologically. Although *C*. *fernambucensis* has been classified as “Least Concern” on the IUCN Red List of Threatened Species, some plant populations have been declining due to habitat loss [9].

Climate change poses a threat to global biodiversity, to which species respond in different ways. In some cacti, lower resilience is exacerbated by anthropogenic pressures, which intensify the loss of genetic diversity [10]. Nevertheless, studies indicate that columnar cacti, especially those with restricted distributions, may be more resilient to genetic erosion than previously thought, although few species have been extensively investigated [11]. In this sense, the assessment of genetic variability is fundamental to understanding the structure and diversity of natural populations, informing kinship relationships, and guiding conservation and genetic improvement programs [12].

The identification of superior genotypes with adaptive and productive traits is fundamental for the development of strategies for the sustainable use of genetic resources [13]. This diversity can be assessed through morphological, physiological, or molecular traits [12]. In particular, the analysis of phenotypic traits related to germination and seedling vigor allows us to understand the intra- and interpopulation genetic structure, providing input for in situ and ex situ conservation strategies, as well as for genetic improvement programs [14].

Despite recent advances, there are still significant gaps in knowledge about genetic variability in natural populations of Cactaceae, especially regarding the relationship between phenotypic traits and underlying genetic diversity [15]. The morphological and physiological traits of fruits and seeds are indirect but effective indicators of genetic variability, as they reflect the expression of genes related to the environmental adaptation and reproductive success of plants [16]. Thus, although it does not use molecular markers, phenotypic characterization can reveal population structure and indicate traits useful for selection and conservation programs. In this context, understanding genetic variability plays a central role in guiding conservation, sustainable management, and breeding efforts, especially in light of the impacts of climate change and habitat fragmentation.

Considering the importance of beach *mandacaru* in *restinga* forests and the need to assess genetic diversity for conservation and genetic improvement purposes, the objective of this study was to assess the phenotypic variability using morphophysiological traits in natural populations of this species in order to identify promising genotypes.

## 2. Materials and Methods

### 2.1. Study Site

**Figure 1:**
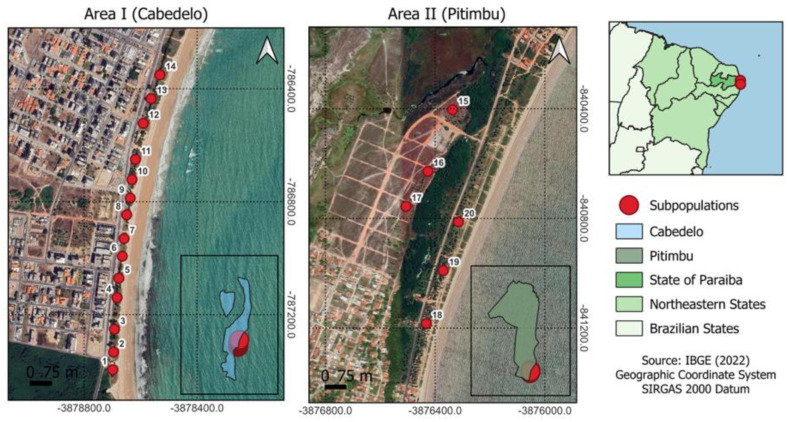
Location of the subpopulations of *Cereus fernambucensis* in *restinga* areas in the municipalities of Cabedelo and Pitimbu, Paraíba, Brazil.

The two populations of *Cereus fernambucensis* evaluated are located in two *restinga* areas in the municipalities of Cabedelo and Pitimbu on the coast of the state of Paraíba, Brazil. The distance between the two municipalities is 54.66 km in a straight line and 72 km by road. The region has a hot and humid tropical climate, with dry and rainy seasons, as well as high temperatures throughout the year. The environmental characteristics of the two areas are described in Table 1. Twenty plant subpopulations were selected in the areas, 14 in area I (Cabedelo) and 6 in area II (Pitimbu) (Figure 1). Subpopulations were formed by clusters of spatially contiguous individuals within the same deme. The geographical distance imposed by physical barriers, such as strips of sand or hedges formed naturally by shrub vegetation, was the criterion established to characterize the subpopulations in each location. The distance between one subpopulation and another in the same population was approximately 30 m.

**Figure 2:**
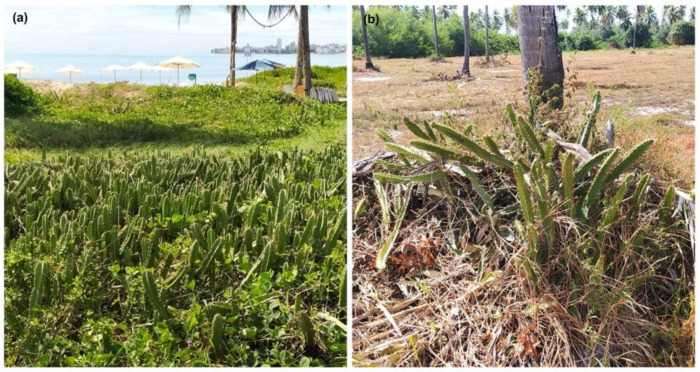
Populations of *Cereus fernambucensis* in *restinga* areas in the municipalities of Cabedelo (**a**) and Pitimbu (**b**), Paraíba, Brazil.

In area I (Cabedelo), there is preserved native *restinga* vegetation, adapted to sandy soil conditions, salinity, and strong sunlight, composed of herbaceous, shrubby, and arboreal species that form mosaics of high biodiversity, in addition to performing ecological functions such as dune fixation, protection against coastal erosion, and shelter for local fauna. In area II (Pitimbu), there is an anthropized *restinga*, where human activity, such as unregulated tourism, deforestation, and pollution, has resulted in the loss of vegetation cover, habitat fragmentation, soil compaction, and greater vulnerability to erosion and invasion by exotic species, compromising the environmental services and resilience of this ecosystem (Figure 2). Cabedelo has a predominance of native forest areas, which occupy 26.7% of the territory, while agriculture and livestock represent 11% of its surface area. Pitimbu has 22.4% native forest cover, but stands out above all for the strong presence of agricultural and livestock activities, which cover 64.7% of the municipality [17].

Data collection took place from August 2023 to November 2024, and fruit harvesting occurred continuously throughout the months, with peaks of fruiting in the hottest months (January to March) in both plant populations. After collection, the fruits were taken to the Seed Analysis Laboratory of the Department of Plant Science at the Center for Agricultural Sciences of the Federal University of Paraíba (DFCA-CCA/UFPB) for biometric and physiological analysis.

### 2.2. Evaluated Traits

#### 2.2.1. Characterization of the Vegetative Structure

For each subpopulation, 20 plants were measured for height, cladode diameter, and number of ribs on each evaluated cladode, which were distributed in four replicates to obtain the means. The cladodes were selected randomly from the subpopulations. As *C*. *fernambucensis* grows in clusters, which makes it difficult to identify the number of individuals and perform evaluations due to the presence of thorns, height was measured from the ground to the apex of the cladode using a tape measure, whereas diameter was measured at the widest base of the cladode using a caliper. Height and diameter measurements were expressed in centimeters, and the number of ribs was expressed in units.

#### 2.2.2. Physical Traits of the Fruit

Forty mature fruits from each plant subpopulation were used for biometric measurements. Length and diameter were measured using a digital caliper (0.01 mm accuracy). Fresh fruit mass was obtained using an analytical balance (0.001 g accuracy), and the number of seeds per fruit was determined via manual dissection and counting. The seeds were washed under running water to remove excess pulp using a fine-mesh sieve and dried on paper towels for 96 h on a laboratory bench at room temperature (25 ± 2 °C and 65 ± 5% RH).

#### 2.2.3. Physical and Physiological Characterization of Seeds

The thousand-seed weight was determined according to the criteria established by the Rules for Seed Analysis [18]. For the germination evaluation, the seeds were sown in four replicates per treatment, consisting of 50 seeds each (*n* = 200 seeds per treatment), which were germinated in paper substrate (on blotting paper), previously sterilized and moistened with distilled water in a volume (mL) equivalent to 2.5 times their dry weight inside transparent acrylic boxes (gerbox) and watered with distilled water when necessary. The material was then transferred to a biological oxygen demand (BOD) germination chamber at a constant temperature of 25 ± 1 °C with a photoperiod of 12 h.

Germinated seeds were counted daily until the 21st day after sowing. The protrusion of a primary root with a length of 1 mm was the criterion for the germination of *C*. *fernambucensis* seeds. The response variables evaluated were the final germination percentage (G%), germination speed index (GSI) [19], and mean germination time (MGT) [20].

The physical and physiological quality parameters of the seeds were calculated using the following equations:

(a) $TSW=\frac{W\times 1000}{N}$

where: *TSW* = Thousand-seed weight (in grams); W = Total weight of the sample (in grams); N = Total number of seeds in the sample.

(b) $G(\%)=\frac{Ng}{Nt}\times 100$

where: *G*(%) = Percentage of germination; *Ng* = Number of germinated seeds; *Nt* = Total number of seeds placed for germination.

(c) $GSI=\frac{G_{1}}{N_{1}}+\frac{G_{2}}{N_{2}}+\frac{G_{3}}{N_{3}}+\cdots +\frac{G_{n}}{N_{n}}$

where: *GSI* = Germination speed index; *G*_1_, *G*_2_, *G*_3_…*G_n_* = Number of seeds germinated in each daily count; *N*_1_, *N*_2_, *N*_3_…*N_n_* = Number of days from sowing to final count.

(d) $MGT=\frac{\sum (G_{i}\times N_{i})}{\sum G_{i}}$

where: *MGT* = Mean germination time (days); *G_i_* = Number of seeds germinated on the day; *N_i_* = Day corresponding to the count.

#### 2.2.4. Seedling Performance

The length and dry mass of the seedlings at the end of the germination test were evaluated, and the total length (SL) of the seedlings was measured with the aid of a ruler, with the results expressed in cm seedling^−1^. After the length was measured, the seedlings were placed in previously identified mono paper bags and placed in an oven with forced air circulation at 65 °C for 72 h to obtain the seedling dry mass (SDM). After drying, the samples were weighed on an analytical balance with an accuracy of 0.001 g, and the results were expressed in mg seedling^−1^. Biomass density (DEN) [21] and the seed vigor index (SVI) [22] were also calculated. Biomass density was determined using dry mass and seedling length, expressed in milligrams per centimeter of seedling (mg cm^−1^), which was calculated via the following equation: DEN = SDM (mg)/SL (cm). The seed vigor index was determined using the following equation: SVI = G (%) × SL (cm).

### 2.3. Data Design and Analysis

The experimental design adopted was completely randomized, with four replicates for each treatment (plant subpopulation), i.e., each treatment totaled 20 plants, 40 fruits, and 200 seeds, ensuring representativeness and independence between replicates. The quantitative data were tested for normality and homoscedasticity of residual variances, followed by analysis of variance, with subsequent grouping of means by the Scott–Knott test at the 5% probability level. The variables analyzed between the two populations of the two areas were represented by boxplots.

The data were standardized (mean = 0; variance = 1) before multivariate procedures; Tocher’s grouping was performed based on generalized Mahalanobis distances (D^2^) [23], which was to maintain internal homogeneity (lowest mean D^2^ within the group) and maximize heterogeneity between groups. The unweighted pair group method with arithmetic mean (UPGMA) was also used. The cutoff point of the generated dendrogram and the number of groups were estimated according to Mojena’s method [24] based on the relative size of the distances in the dendrogram.

To estimate the relative contribution of traits according to Singh [25] for each trait, the sum of standardized differences between all pairs of genotypes was calculated, expressed as a fraction of the total sum obtained for all traits. The values were then converted into percentages, indicating the relative contribution of each variable to the formation of Mahalanobis generalized distances (D^2^). Next, a selection index was applied that considers the adjusted phenotypic means and the distances of each individual from an ideal genotype (genotype–ideotype distance index), a selection technique that allows for the identification and selection of ideal genotypes.

Genetic parameters and their estimators were analyzed for each trait via the following mathematical expressions [26]:

(a) Phenotypic variance: ${\hat{\sigma }}_{f}^{2}=\frac{{QM}_{g}}{k}$
(b) Environmental variance: ${\hat{\sigma }}_{e}^{2}=\frac{{QM}_{r}}{k}$
(c) Genetic variance: ${\hat{\sigma }}_{g}^{2}=\frac{{QM}_{g}-{QM}_{r}}{k}$

where *Q*Mg and *QM*r correspond to the mean squares of the genotype and error, respectively, and *k* is the number of replicates. From these components, the following genetic parameters were estimated:

(d) Broad-sense repeatability estimates: $h^{2}=\frac{{\hat{\sigma }}_{g}^{2}}{{\hat{\sigma }}_{f}^{2}}$
(e) Coefficient of genetic variation: ${CV}_{g}=\frac{\sqrt{\sigma_{g}}}{m}\times 100$
(f) Coefficient of environmental variation: ${CV}_{e}=\frac{\sqrt{\sigma_{r}}}{m}\times 100$
(g) Ratio $\frac{{CV}_{g}}{{CV}_{e}}$

Statistical analyses were performed via Genes software (version 1990.2023.15) [27] and R version 4.2.1 [28] via the ScottKnott [29], candisc [30], biotools [31], and factoextra [32] packages.

## 3. Results

For 12 of the 15 traits evaluated, there was a significant effect at a 1% and 5% probability (Table 2), indicating the existence of genetic variability among the genotypes evaluated. The diameter of the cladode and fruit, as well as the fresh weight of the fruit, were the only variables whose treatment effects were not significant. The values of broad-sense repeatability estimates (h^2^) ranged from 0 to 96.2%, with the lowest values observed for the above-mentioned variables where the data were not significant. In contrast, the highest values of broad-sense repeatability estimates were observed for traits related to germination speed, such as the mean germination time (MGT) and germination speed index (GSI), with values of 96.20 and 94.17%, respectively.

The ratio between the genetic and environmental coefficients of variation (CVg/CVe) was greater than 1 for five traits: thousand-seed weight, germination speed index, mean germination time, seedling length, and the seed vigor index (Table 2), indicating a favorable situation for selection. The coefficients of variation (CV) of the experiment ranged from 3.08 to 54.76%, with the highest values recorded for fresh fruit weight, number of seeds per fruit, and plant height, with values of 54.76, 52.03, and 25.12%, respectively. This significant variation is due to the range of data for these traits. In contrast, the CV values for the other traits were less than 20%.

**Figure 3:**
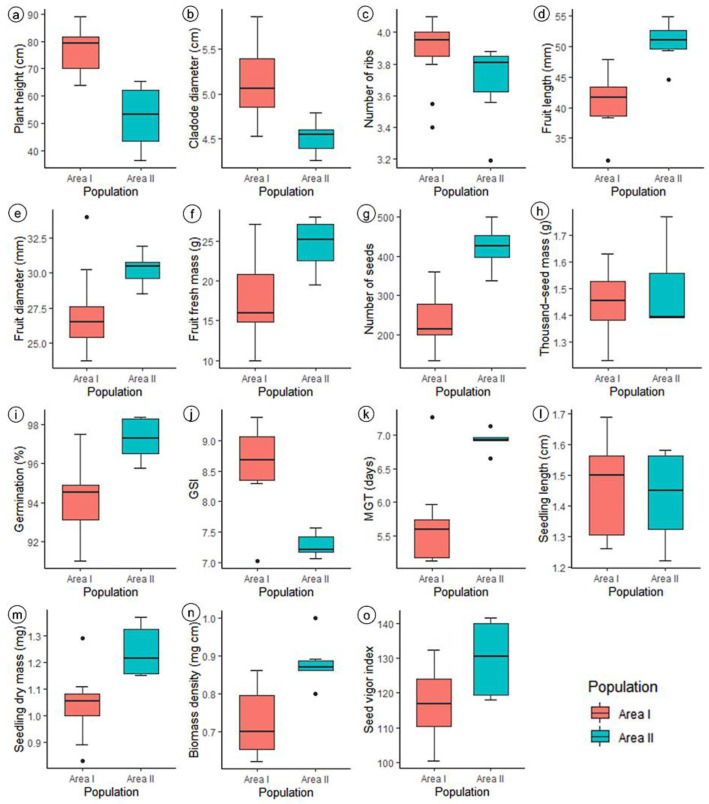
Boxplot analysis of the phenotypic traits of *Cereus fernambucensis* from two natural plant populations in the *restinga* of Cabedelo (area I) and Pitimbu (area II), Paraíba, Brazil. (**a**) plant height (cm); (**b**) cladode diameter (cm); (**c**) number of ribs; (**d**) fruit length (mm); (**e**) fruit diameter (mm); (**f**) fresh fruit weight (g); (**g**) number of seeds; (**h**) thousand-seed weight (g); (**i**) germination percentage; (**j**) germination speed index (GSI); (**k**) mean germination time (MGT—days); (**l**) seedling length (cm); (**m**) seedling dry weight (mg seedling^−1^); (**n**) biomass density (mg cm seedling^−1^); (**o**) seed vigor index. The narrowest portion within each box represents the median; box notch boundaries indicate the median confidence interval; lower and upper bounds of the box show the lower and upper quartiles, respectively; vertical bars represent the maximum and minimum values; isolated points outside these limits are considered outliers.

Considering the results obtained in the Scott–Knott test at a 5% probability (Table S1—Supplementary Materials), the plant subpopulations were grouped into four classes, varying according to the characteristic analyzed. A boxplot analysis of the phenotypic traits of the two populations of *C*. *fernambucensis* (Figure 3) revealed that, in general, the plants in area I exhibited superior vegetative traits compared to plants in area II, such as height and cladode diameter. In contrast, plants in area II, although shorter, stood out for having larger fruits with more seeds and greater mass. With respect to seeds, the plant population in area II showed more efficient germination in terms of germination percentage. On the other hand, seeds from area I showed faster germination onset, but seedlings from area II demonstrated greater vigor in terms of dry mass and biomass density.

The Tocher optimization method, which is based on the Mahalanobis distance, allowed the subpopulations of plants studied to be separated into nine groups (Table 3), demonstrating that there is high variability among plants in terms of the traits evaluated. Groups I and IV were formed by two plant subpopulations each from area I, whereas groups II and III, formed exclusively by plants from area II, together accounted for 45% of the subpopulations, with 6 and 3, respectively. Groups V and VI also contained two subpopulations each, the latter including plants from both populations, and groups VII, VIII, and IX were composed of a single plant subpopulation each.

**Figure 4:**
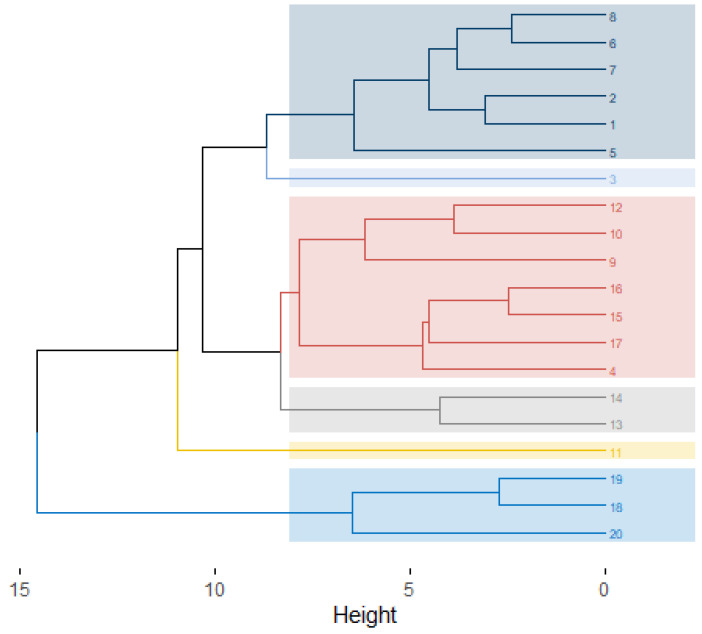
Dendrogram generated via UPGMA (standardized Euclidean distance) based on 15 phenotypic traits of *Cereus fernambucensis*. Cutoff point = 10.23 (Mojena’s method) groups genotypes into 6 clusters (distinct colors). Genotype numbering: 1–14 = area I, 15–20 = area II.

The dendrogram generated by the UPGMA (Unweighted Pair Group Method with Arithmetic Mean) clustering method, using standardized Euclidean distance, is shown in Figure 4. According to Mojena’s method [24], the dendrogram cutoff point was set at 10.23, resulting in the formation of six groups, each represented by a different color. Of these, two groups were composed of only one genotype: genotypes 3 and 11, both from the plant population in area I. A difference was observed between the Tocher and UPGMA methods in the number of groups formed, with the former generating nine groups and the latter resulting in only six. However, it is important to note that genotype 11 was grouped separately in both methods, forming a single group (Table 3; Figure 4).

**Figure 5:**
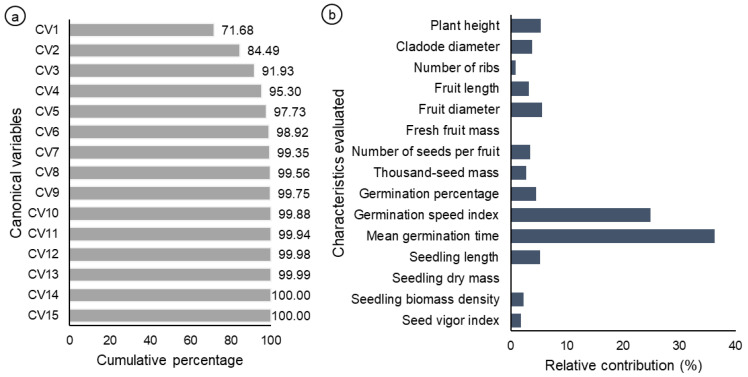
(**a**) Analysis of weighting coefficients obtained by canonical variables from eigenvalue estimates. (**b**) Estimates of the relative contribution of each variable to genetic divergence on the basis of Mahalanobis distances, according to Singh’s criterion.

Analysis of the canonical variables revealed that the first three accumulated variables explained more than 90% of the total variation (Figure 5a), and Figure 5b shows the relative importance of the 15 traits evaluated. According to Singh’s method [25], two of these variables accounted for 61% of the total dissimilarity, whereas the others contributed 39% (Figure 5b). Among the variables analyzed, the mean germination time (36.26%) and the germination speed index (24.80%) were the most significant in explaining the dissimilarity between the subpopulations of plants studied. These two variables showed a strong negative correlation, since they are inversely proportional (Table S2—Supplementary Materials). On the other hand, the variables that contributed little or nothing to the divergence were the fresh fruit mass and dry seedling mass, both with values of 0%, followed by the number of ribs, which contributed only 0.8%. These results indicate that specific traits are more efficient at explaining the dissimilarity observed between plant populations.

According to the selection index based on genotype–ideotype distance, six plant subpopulations, two of which originated from the *C*. *fernambucensis* plant population in area I (4 and 13) and four from area II (15, 16, 17, and 18), were selected as the most promising for breeding studies. In general, by selecting individuals belonging to these plant subpopulations, it is possible to obtain gains in fruit length, number of seeds per fruit, and seed and seedling vigor (Table S3—Supplementary Materials).

## 4. Discussion

This study investigated the phenotypic variability between and within natural populations of *Cereus fernambucensis* via 15 quantitative variables related to the morphophysiological traits of plants and propagules. Our results indicated that the phenotypic traits of *C*. *fernambucensis* were able to express the high genetic diversity in the subpopulations analyzed, suggesting the selection of the most promising ones and the traits that contributed the most to this dissimilarity. This finding adds to the growing body of data on genetic variability in Cactaceae, showing that individuals in this family, especially those with columnar growth, have high levels of genetic variability [11,33].

Cacti constitute one of the most threatened taxonomic groups in the world, with approximately 31% of species assessed as at risk, with indiscriminate collection and agriculture being the main factors of local extinctions [34]. Therefore, it is necessary to understand how prepared xerophytic plant species face ecosystem degradation and habitat fragmentation, considering the levels of genetic diversity present in plant populations [11]. Although the genetic variation of columnar cacti with wide geographical distribution is high, in species with restricted distribution, such as those found in eastern Brazil, there are varying patterns of genetic variability [33]. Several factors can affect genetic diversity in plants, including reproductive biology and pollinator activity, which determine patterns of mating and gene dispersal, as well as geographic distribution, which plays a crucial role in genetic variation between and within plant populations [35].

In accordance with Tocher’s grouping method, when individuals of *C*. *fernambucensis* are selected for fruit and seed collection in breeding and conservation programs, it is important to prioritize subpopulations of plants from distinct groups, especially those with desirable traits, since combining different groups can increase variability and result in significant genetic gains [36,37]. On the other hand, *C*. *fernambucensis* fruits harvested from subpopulations of plants belonging to the same group can, in principle, constitute the same seed lot since they are similar in terms of quality.

The Tocher clustering method also enabled the comparison of results and the identification of subpopulations of *C*. *fernambucensis* with similar and divergent behaviors. This grouping revealed that at least one subpopulation of plants from both locations was classified into group VI (Table 3). These results corroborate those of the study by Bispo et al. [36], who, when studying the genetic diversity of 20 genotypes of *Mauritia flexuosa* L.f. (Arecaceae) via Tocher’s optimization method on the basis of fruit and seed traits, identified two groups formed by parent plants from different origins.

In a study with *Tacinga inamoena* (K Schum.) NP Taylor & Stuppy (Cactaceae), Lima et al. [15] assessed genetic diversity through morphological, physicochemical traits, and RAPD markers in three plant populations in the Brazilian semiarid region, indicating significant variability and highlighting the plant population of Assú, Rio Grande do Norte, as a priority for genetic improvement and conservation. According to Hamrick and Godt [38], the high genetic variability in several plant species can be attributed to characteristics such as perennial life forms. Both *T*. *inamoena* and *C*. *fernambucensis* reproduce vegetatively through detached stems, which is characterized as a long-lasting perennial life form. This characteristic allows individuals in Cactaceae populations to persist for several generations [33], ensuring survival even under adverse environmental conditions. However, in *C*. *fernambucensis*, sexual reproduction by seeds also plays a fundamental role, as in addition to contributing to the formation of new individuals, it ensures the maintenance and increase in genetic variability, strengthening the adaptive capacity of the species over time.

All the traits evaluated are important in the study of divergence among individuals of *C*. *fernambucensis*; however, the traits that contribute least to genetic diversity in this type of study can be discarded. Fruit fresh weight, seedling dry weight, and number of ribs were the variables that contributed least to the original variability of the data, and consequently to the cluster analysis. In this sense, recognizing the importance and relative contribution of traits to genetic divergence enables the more efficient planning of future studies, allowing low-relevance traits to be disregarded and focusing only on those that have a significant contribution, which reduces the work and costs of experiments [39,40].

The results show that the divergence between the subpopulations of *C*. *fernambucensis* is generally based on the physiological quality of the seeds, a characteristic also observed in other tropical plant species [40,41]. The differences in seed physiological quality and seedling vigor among the plant populations in areas I and II can be attributed to environmental influences and genetic variability, in agreement with the findings of Silva et al. [41] and Bezerra et al. [42], who reported that these influences promote variations in the intrinsic traits of seeds within the same species. The evaluation of seed quality, carried out through germination and vigor tests, is essential for predicting field performance [43], determining genetic variability, and differentiating the quality of seeds from different parent plants [39,42].

Genetic diversity is essential in the selection and evolution of species because it ensures resistance to pests and diseases, enables adaptations to biotic and abiotic stresses, and helps maintain desirable traits in crops, while plant populations with low diversity become vulnerable [44]. According to these authors, natural and artificial selection influence the phenotypic traits of plant species, whose expression depends on genotype–environment interactions, with the effectiveness of artificial selection being conditioned by the availability of genetic variation in the plant population. However, in recent years, the genetic diversity of wild plant populations has been declining globally [45], mainly due to reductions in geographical areas, infrastructure development, climate change, habitat fragmentation, population decline, overgrazing, and a lack of adequate management and conservation practices [46,47,48].

The loss of genetic variability in native plant populations influences the adaptive and evolutionary processes of species [37]. However, columnar cacti are characterized by high levels of genetic variability [11], and in the genus *Cereus*, there are high levels of genetic diversity in South America [49]. This high genetic variability in columnar cacti occurring across a wide latitudinal range indicates that these species may be more resilient to genetic erosion caused by habitat fragmentation [11], as exemplified by *C*. *fernambucensis*. Studies such as this are essential for gains in plant breeding through selection, and the *C*. *fernambucensis* populations analyzed have potential in this regard, with good genetic diversity both within and between populations, as evidenced by the diversity of groups formed.

Although the loss of genetic variability can occur naturally as a result of the selection of more adaptive alleles, this process is not always harmful, as it contributes to improving population fitness by eliminating disadvantageous variants. However, when the reduction in genetic variability results from non-selective factors, such as habitat fragmentation, genetic drift, or population size reduction, the effects tend to be negative, as they limit the adaptive and evolutionary potential of populations [50]. Thus, maintaining an adequate level of genetic diversity is essential to ensure the resilience of native plant species in the face of environmental changes and future selective pressures [16].

The difference observed in fruit size and number of seeds between populations in areas I and II may be associated, among other factors, with variations in pollination dynamics. Although this study did not directly evaluate the pollinator community, more altered environments often show changes in resource availability and floral visitation patterns, which could influence the amount of pollen deposited, and consequently, reproductive success. Thus, it is possible that differences in the abundance or efficiency of pollinators, although not measured here, play some role in the contrasts observed between the areas. However, such interpretations remain hypothetical and should be confirmed in future studies that include specific data on the composition and activity of pollinator communities in the regions analyzed.

From a conservation perspective, maintaining genetic variability is essential to ensure the resilience and adaptation of populations over time. In this context, seed propagation is a fundamental strategy, especially for native species in natural ecosystems. Thus, evaluating seed vigor and seedling performance is an indispensable step in selecting parents with greater adaptive potential, guiding conservation actions, and supporting programs for the recovery of degraded areas. The results obtained in this study provide a solid basis for guiding future stages of genetic improvement programs for this species, with an emphasis on identifying divergent and promising genotypes for broader agronomic evaluations, including traits such as fruit productivity, quality, and viability for vegetative propagation.

## 5. Conclusions

The high genetic diversity in natural populations of *Cereus fernambucensis* confirms its potential for strategic targeting in the collection of genetic material, which is essential for in situ and ex situ conservation programs and for the development of effective genetic improvement strategies.

The mean germination time and seed germination speed indices stand out as the most relevant phenotypic traits for explaining phenotypic variability among genotypes and may serve as key criteria in the selection of superior individuals for preservation and use.

The genetic divergence observed, both intra- and interpopulation, indicates the need to include representative samples from different genetic groups to capture the total genetic variability of the species, ensuring the maintenance of its adaptive potential.

Although habitat fragmentation and loss in natural areas indicate risks of inbreeding and increased phenotypic uniformity in the plant population in area II, the high phenotypic variability identified reveals genetic resilience that can support the conservation and sustainable management of this population.

These results represent valuable contributions to the knowledge of the genetic resources of *C*. *fernambucensis*, providing a basis for future genetic conservation actions, germplasm bank management, and breeding programs that promote the sustainability and evolutionary resilience of the species.

Our findings provide quantitative evidence supporting the inclusion of *C. fernambucensis* in integrated coastal conservation frameworks, linking phenotypic diversity with adaptive resilience under ongoing climate change.

## Figures and Tables

**Table 1:** Environmental characteristics of two natural populations of *Cereus fernambucensis* in *restinga* areas in the municipalities of Cabedelo and Pitimbu, Paraíba, Brazil.

Plant Population	Municipality	Altitude (masl.)	Mean Temperature (min.–max.) (°C)	Mean Annual Precipitation (mm)	Landscape Conservation Status
Area I	Cabedelo	2.5	25.8 (22–30)	1170	Preserved *restinga*
Area II	Pitimbu	2.5	25.9 (21–30)	1165	Anthropized *restinga*

**Table 2:** Analysis of variance of the phenotypic traits of the vegetative structure of plants, fruits, seeds, and seedlings of *Cereus fernambucensis* from 20 plant subpopulations in two *restinga* areas in Paraíba, Brazil.

**Variation Factor**	**Mean Squares**				
	**PH (cm)**	**CD (cm)**	**NR**	**FL (mm)**	**FD (mm)**
Genotypes	856.78 **	0.78 ^ns^	0.3 **	136.38 *	30.45 ^ns^
Residue	303.77	0.86	0.10	66.76	20.66
h^2^ (%)	64.54	0.0	63.88	51.04	32.14
Ve	75.94	0.21	0.02	16.69	5.16
Vg	138.25	0.0	0.04	17.40	2.44
CVg/CVe	0.67	0.0	0.66	0.51	0.34
CV (%)	25.12	18.87	8.54	18.58	16.27
**Variation Factor**	**Mean Squares**				
	**FFM (g)**	**NS**	**TSM (g)**	**G (%)**	**GSI**
Genotypes	118.85 ^ns^	46,075.81 *	0.07 **	19.42 **	2.70 **
Residue	116.73	23251.43	0.007	8.59	0.15
h^2^ (%)	1.78	49.53	89.12	55.75	94.17
Ve	29.18	5812.85	0.0	2.14	0.03
Vg	0.53	5706.09	0.01	2.70	0.63
CVg/CVe	0.06	0.50	1.43	0.56	2.01
CV (%)	54.76	52.03	6.02	3.08	4.81
**Variation Factor**	**Mean Squares**				
	**MGT (dias)**	**SL (cm)**	**SDM (mg)**	**DEN** **(cm mg^−1^)**	**SVI**
Genotypes	2.37 **	0.08 **	0.08 **	0.04 **	544.28 **
Residue	0.09	0.01	0.01	0.01	78.64
h^2^ (%)	96.20	83.42	79.59	74.73	85.55
Ve	0.02	0.0	0.0	0.0	19.66
Vg	0.57	0.01	0.01	0.0	116.41
CVg/CVe	2.51	1.12	0.98	0.86	1.21
CV (%)	5.0	8.13	11.74	14.45	7.32

* Significant effect at 1% (**), at 5% (*) and non–significant effect (^ns^) by the F test. Caption: Broad-sense repeatability estimates (h^2^); Environmental variance (Ve); Genetic variance (Vg); ratio between genetic and environmental coefficient of variation (CVg/CVe); coefficient of variation (CV); plant height (PH); cladode diameter (CD); number of ribs (NR); fruit length (FL); fruit diameter (FD); fruit fresh mass (FFM); number of seeds per fruit (NS); thousand-seed mass (TSM); germination percentage (G); germination speed index (GSI); mean germination time (MGT); seedling length (SL); seedling dry mass (SDM); biomass density (DEN); seed vigor index (SVI).

**Table 3:** Clustering of 20 subpopulations of *Cereus fernambucensis* plants via the Tocher optimization method, based on the standardized mean Euclidean distance estimated from 15 quantitative traits of plants, fruits, seeds, and seedlings.

Groups	Plant Subpopulations ^1^
I	15–16
II	2–7–8–4–6–5
III	13–14–12
IV	18–19
V	1–9
VI	3–17
VII	20
VIII	11
IX	10

^1^ Identification of plant subpopulations and their respective locations: 1 to 14 (area I), 15 to 20 (area II).

## Data Availability

The original contributions presented in the study are included in the article. The data presented in this study are available on request from the corresponding author.

## Supplementary Materials

The following supporting information can be downloaded at: https://www.mdpi.com/article/10.3390/biology14121702/s1, Table S1: Averages of the quantitative traits related to the vegetative structure of mother plants, fruit size, seed physiological quality, and seedling vigor of *Cereus fernambucensis* in 20 plant subpopulations in *Restinga* areas, Paraíba, Brazil. Table S2: Correlations between the phenotypic traits of *Cereus fernambucensis* Lem. (Cactaceae). Table S3: Estimates of percentage gains (GS%) for fifteen traits in *Cereus fernambucensis* Lem. (Cactaceae) using selection by genotype–ideotype index.

## Abbreviations

The following abbreviations are used in this manuscript:

BAF	Brazilian Atlantic Forest
BOD	Biological oxygen demand
CAM	Crassulacean acid metabolism
CCA	Centro de Ciências Agrárias
CD	Cladode diameter
CV	Coefficient of variation
CVe	Environmental coefficient of variation
CVg	Genetic coefficient of variation
DEN	Biomass density
DFCA	Departamento de Fitotecnia e Ciências Ambientais
FD	Fruit diameter
FFM	Fruit fresh mass
FL	Fruit length
G%	Germination percentage
GSI	Germination speed index
IUCN	International Union for Conservation of Nature
MGT	Mean germination time
NR	Number of cladode ribs
NS	Number of seeds per fruit
PH	Plant height
RAPD	Random Amplified Polymorphic DNA
RH	Relative humidity
SDM	Seedling dry mass
SL	Seedling length
SVI	Seed vigor index
TSM	Thousand-seed mass
UFPB	Universidade Federal da Paraíba
UPGMA	Unweighted Pair Group Method with Arithmetic Mean
