# Supplementary material for: Analysis of Phenotypic Variability in Natural Populations of Cereus fernambucensis Lem. (Cactaceae)
Source: Biology (Basel). 2025 Nov 29;14(12):1702. doi: 10.3390/biology14121702 (PMC12730033; doi:10.3390/biology14121702)
Supplement: Supplementary file 1 [file biology-14-01702-s001.zip › Table S3 - Supplementary Material.pdf]

Supplementary Material

Table S3: Estimates of percentage gains (GS%) for fifteen traits in *Cereus fernambucensis* Lem. (Cactaceae) using selection by genotype–ideotype index.

Traits	Genotype–ideotype distance index	
	**GS%**	**Selected genotypes**
Plant height (cm)	–3.05	
Cladode diameter (cm)	0.0	
Number of ribs	2.49	
Fruit length (mm)	4.65	
Fruit diameter (mm)	2.86	
Fresh fruit mass (g)	0.39	
Number of seeds per fruit	14.4	
Thousand–seed mass (g)	4.01	4 – 13 – 15 – 16 – 17 – 18
Germination percentagem (%)	0.42	
Germination speed index	–5.05	
Mean germination time (days)	6.91	
Seedling length (cm)	–2.04	
Seedling dry mass (mg)	10.12	
Seedling biomass density (cm mg^{-1})	11.58	
Seed vigor index	4.16	
