# Supplementary material for: Analysis of Phenotypic Variability in Natural Populations of Cereus fernambucensis Lem. (Cactaceae)
Source: Biology (Basel). 2025 Nov 29;14(12):1702. doi: 10.3390/biology14121702 (PMC12730033; doi:10.3390/biology14121702)
Supplement: Supplementary file 1 [file biology-14-01702-s001.zip › Table S1 - Supplementary Material.pdf]

Supplementary Material

Table S1. Averages of the quantitative traits related to the vegetative structure of mother plants, fruit size, seed physiological quality and seedling vigor of *Cereus fernambucensis* in 20 plant subpopulations in *Restinga* areas, Paraíba, Brazil.

Subpop*	Plant height (cm)	Plant diameter (mm)	Number of ribs	Fruit length (mm)	Fruit diameter (mm)	Fruit weight (g)	Number of seeds per fruit	Thousand-seed mass (g)	Germination (%)	GSI	MGT (days)	Seedling length (cm)	Seedling dry mass (mg)	Biomass density (mg cm^{-1})	Seed vigor index
**1**	64.35 a	50.89 a	3.85 a	31.61 b	26.73 a	14.78 a	198 b	1.39 c	94 b	8.79 a	5.49 b	1.62 a	1.00 b	0.61 b	122.6 b
**2**	82.30 a	51.68 a	4.00 a	38.61 b	25.87 a	20.90 a	280 b	1.45 c	95 b	8.56 b	5.67 b	1.50 a	0.99 b	0.66 b	118.5 c
**3**	72.80 a	50.11 a	4.05 a	43.51 b	27.71 a	16.07 a	215 b	1.22 d	98 a	7.02 c	7.26 a	1.25 b	0.88 c	0.70 b	104.4 c
**4**	80.40 a	50.34 a	3.95 a	41.31 b	27.37 a	19.27 a	258 b	1.62 b	95 b	8.44 b	5.86 b	1.51 a	1.29 a	0.85 a	132.4 a
**5**	89.10 a	46.25 a	3.85 a	31.35 b	24.21 a	9.96 a	133 b	1.53 b	95 b	8.55 b	5.72 b	1.39 b	1.04 b	0.74 b	115.2 c
**6**	84.15 a	46.97 a	3.85 a	38.33 b	26.29 a	15.82 a	212 b	1.61 b	94 b	8.30 b	5.96 b	1.68 a	1.07 b	0.63 b	129.8 b
**7**	80.55 a	56.30 a	3.95 a	41.94 b	26.85 a	15.56 a	209 b	1.45 c	93 b	8.31 b	5.73 b	1.59 a	1.04 b	0.65 b	122.7 b
**8**	81.90 a	48.24 a	3.95 a	38.74 b	25.23 a	14.92 a	200 b	1.47 c	92 b	8.29 b	5.71 b	1.49 a	0.92 c	0.62 b	110.6 c
**9**	63.80 a	49.43 a	3.55 b	44.21 b	28.01 a	20.90 a	280 b	1.52 b	95 b	9.28 a	5.12 c	1.56 a	1.07 b	0.68 b	124.4 b
**10**	86.30 a	54.49 a	3.80 a	41.12 b	23.75 a	11.43 a	153 b	1.60 b	97 a	9.37 a	5.19 c	1.54 a	1.10 b	0.72 b	127.8 b
**11**	67.60 a	45.31 a	3.40 b	42.09 b	24.59 a	25.22 a	338 a	1.27 d	96 a	8.97 a	5.45 b	1.27 b	0.82 c	0.64 b	100.4 c
**12**	78.15 a	58.61 a	4.10 a	42.78 b	26.11 a	14.82 a	198 b	1.45 c	95 b	9.29 a	5.14 c	1.32 b	1.06 b	0.80 a	113.1 c
**13**	81.10 a	52.30 a	4.00 a	47.92 a	34.00 a	27.06 a	361 a	1.38 d	93 b	9.08 a	5.15 c	1.29 b	1.07 b	0.83 a	110.4 c
**14**	73.85 a	55.42 a	4.10 a	44.69 b	30.22 a	20.62 a	276 b	1.31 c	91 b	8.86 a	5.16 c	1.27 b	1.09 b	0.85 a	107.8 c
**15**	60.50 a	46.10 a	3.81 a	51.53 a	30.81 a	15.28 a	446 a	1.77 a	97 a	7.48 c	6.92 a	1.58 a	1.35 a	0.85 a	141.6 a
**16**	62.75 a	47.93 a	3.85 a	49.36 a	30.59 a	25.04 a	407 a	1.60 b	97 a	7.56 c	6.65 a	1.54 a	1.36 a	0.89 a	140.6 a
**17**	75.30 a	45.12 a	3.81 a	44.61 b	29.34 a	19.46 a	338 a	1.39 c	96 a	7.16 c	6.92 a	1.31 b	1.15 a	0.88 a	118.2 c
**18**	46.37 b	42.56 a	3.87 a	53.01 a	30.36 a	25.23 a	457 a	1.38 c	98 a	7.06 c	7.12 a	1.21 b	1.18 a	1.00 a	117.9 c
**19**	36.50 b	43.55 a	3.56 b	50.49 a	28.55 a	21.72 a	393 a	1.40 c	98 a	7.22 c	6.96 a	1.35 b	1.15 a	0.85 a	123.1 b
**20**	42.50 b	45.78 a	3.18 b	54.93 a	31.92 a	27.68 a	501 a	1.39 c	98 a	7.19 c	6.92 a	1.56 a	1.24 a	0.80 a	137.9 a
**CV (%)**	25.12	18.87	8.54	18.58	16.27	54.76	52.03	6.02	3.08	4.81	5.0	8.13	11.74	14.45	7.32

* Subpopulations of plants: 1–14 (Area I); 15–20 (Area II). Averages followed by the same letter in the column do not differ by the Scott-Knott test at 5% probability. GSI: germination speed index; MGT: mean germination time. CV: coefficient of variation.
