# Supplementary material for: Analysis of Phenotypic Variability in Natural Populations of Cereus fernambucensis Lem. (Cactaceae)
Source: Biology (Basel). 2025 Nov 29;14(12):1702. doi: 10.3390/biology14121702 (PMC12730033; doi:10.3390/biology14121702)
Supplement: Supplementary file 1 [file biology-14-01702-s001.zip › Table S2 - Supplementary Material.pdf]

## Supplementary Material

**Table S2.** Correlations between the phenotypic traits of *Cereus fernambucensis* Lem. (Cactaceae).

| Traits | CD | NR | FL | FD | FFM | NS | TSM | G | GSI | MGT | SL | SDM | DEN | SVI |
|---|---|---|---|---|---|---|---|---|---|---|---|---|---|---|
| **PH** | 0.60 | 0.47 | –0.86 | –0.55 | –0.83 | –1.00** | 0.23 | –0.77 | 0.64 | –0.64 | 0.15 | –0.36 | –0.45 | –0.28 |
| **CD** | | 0.41 | –0.38 | –0.24 | –0.57 | –0.73 | 0.00 | 0.69 | 0.70 | –0.73 | 0.12 | –0.18 | –0.25 | –0.24 |
| **NR** | | | –0.24 | –0.04 | –0.37 | –0.43 | 0.16 | –0.38 | 0.04 | –0.06 | –0.39 | 0.01 | 0.29 | –0.29 |
| **FL** | | | | 0.83 | 0.87 | 0.97 | –0.50 | 0.66 | –0.57 | 0.59 | 0.19 | 0.63* | 0.76 | 0.39 |
| **FD** | | | | | 0.85 | 0.85 | –0.04 | 0.20 | –0.44 | 0.40 | –0.18 | 0.59 | 0.70 | 0.28 |
| **FFM** | | | | | | 0.98 | –0.03 | 0.41 | –0.42 | 0.42 | –0.19 | 0.51 | 0.62 | 0.27 |
| **NS** | | | | | | | –0.02 | 0.65 | –0.66 | 0.67 | –0.17 | 0.62 | 0.78 | 0.39 |
| **TSM** | | | | | | | | 0.04 | 0.08 | –0.03 | 0.71 | 0.68 | 0.11 | 0.81 |
| **G** | | | | | | | | | –0.69 | 0.79 | –0.14 | 0.44 | 0.55 | 0.39 |
| **GSI** | | | | | | | | | | –0.99** | 0.15 | –0.39 | –0.49 | –0.28 |
| **MGT** | | | | | | | | | | | –0.14 | 0.41 | 0.49 | 0.32 |
| **SL** | | | | | | | | | | | | 0.29 | –0.52 | 0.73 |
| **SDM** | | | | | | | | | | | | | 0.72 | 0.86 |
| **DEN** | | | | | | | | | | | | | | 0.25 |

**, *: Significant at 1% and 5%, respectively, using the bootstrap method with 1,000 simulations. Plant height (PH); cladode diameter (CD); number of ribs (NR); fruit length (FL); fruit diameter (FD); fruit fresh mass (FFM); number of seeds per fruit (NS); thousand–seed mass (TSM); germination percentage (G); germination speed index (GSI); mean germination time (MGT); seedling length (SL); seedling dry mass (SDM); biomass density (DEN); seed vigor index (SVI).
